# Foreign Body Reaction to Implanted Biomaterials and Its Impact in Nerve Neuroprosthetics

**DOI:** 10.3389/fbioe.2021.622524

**Published:** 2021-04-15

**Authors:** Alejandro Carnicer-Lombarte, Shao-Tuan Chen, George G. Malliaras, Damiano G. Barone

**Affiliations:** ^1^Electrical Engineering Division, Department of Engineering, University of Cambridge, Cambridge, United Kingdom; ^2^Division of Neurosurgery, Department of Clinical Neurosciences, University of Cambridge, Cambridge, United Kingdom

**Keywords:** foreign body reaction, nerve neuroprosthetics, neural implants, neural interface, peripheral nerve stimulation, biocompatibility

## Abstract

The implantation of any foreign material into the body leads to the development of an inflammatory and fibrotic process—the foreign body reaction (FBR). Upon implantation into a tissue, cells of the immune system become attracted to the foreign material and attempt to degrade it. If this degradation fails, fibroblasts envelop the material and form a physical barrier to isolate it from the rest of the body. Long-term implantation of medical devices faces a great challenge presented by FBR, as the cellular response disrupts the interface between implant and its target tissue. This is particularly true for nerve neuroprosthetic implants—devices implanted into nerves to address conditions such as sensory loss, muscle paralysis, chronic pain, and epilepsy. Nerve neuroprosthetics rely on tight interfacing between nerve tissue and electrodes to detect the tiny electrical signals carried by axons, and/or electrically stimulate small subsets of axons within a nerve. Moreover, as advances in microfabrication drive the field to increasingly miniaturized nerve implants, the need for a stable, intimate implant-tissue interface is likely to quickly become a limiting factor for the development of new neuroprosthetic implant technologies. Here, we provide an overview of the material-cell interactions leading to the development of FBR. We review current nerve neuroprosthetic technologies (cuff, penetrating, and regenerative interfaces) and how long-term function of these is limited by FBR. Finally, we discuss how material properties (such as stiffness and size), pharmacological therapies, or use of biodegradable materials may be exploited to minimize FBR to nerve neuroprosthetic implants and improve their long-term stability.

## Introduction

Implantable devices constitute a class of treatments for disease or dysfunction with unique therapeutic potential. By remaining implanted for long periods of time while delivering a therapy directly into the affected tissue, implants can target the source of a dysfunction with great accuracy and for as long as required. One of the most versatile class of implants are nerve neuroprosthetics: implantable devices which interface with the peripheral nerves of the body. Nerves serve as communication bridges between the brain and all peripheral structures, transmitting information between the two. Whether by activating nerves through electrical stimulation or by reading their electrical signals, nerve neuroprosthetics offer a myriad of applications such as the control of bladder function (Brindley et al., 1982), management of depression (Nemeroff et al., 2006), and restoration of sensory perception in amputees (D'Anna et al., 2019). While implantable technologies—and in particular nerve neuroprosthetics—have extensively evolved over the last few decades by drawing on advancements in biomaterials and manufacturing techniques, the clinical use of implants continues to be severely hampered by the challenges arising from the hostile environment of the body.

The aim of this review is to highlight one of the major challenges to the clinical translation of implantable materials and devices: the foreign body reaction (FBR). The slow onset of this reaction triggered by the body and its dynamic profile (beginning with an acute inflammatory attack and transitioning to a long-term fibrotic response) make it difficult to predict and test for during the design and development of implantable devices. Research often focuses on the optimisation of implantable technologies for good function over a period of days to weeks after implantation. While it is impractical to test new technologies for longer periods of time at every step, clinical devices have to remain implanted in human patients for years or decades. In order to facilitate bench-to-bedside translation of new technologies, it is therefore crucial to consider the effects that FBR will have on both the implanted device and the surrounding tissue.

In this review we present an overview of the cellular events leading to and maintaining FBR in order to provide an understanding of the processes involved in this reaction and the challenges presented to implanted devices, and discuss some of the general design principles that can be followed to minimize the impact of FBR. We discuss the impact of FBR in the emerging field of nerve neuroprosthetics—how nerve neuroprosthetic designs have evolved over the years and addressed the challenges posed by FBR. Finally, we look at the future of the field by discussing novel strategies being developed to combat FBR to implants, and how these may be implemented in nerve neuroprosthetic devices.

## Implantation of Biomaterials and the Foreign Body Reaction

**Figure 1:**
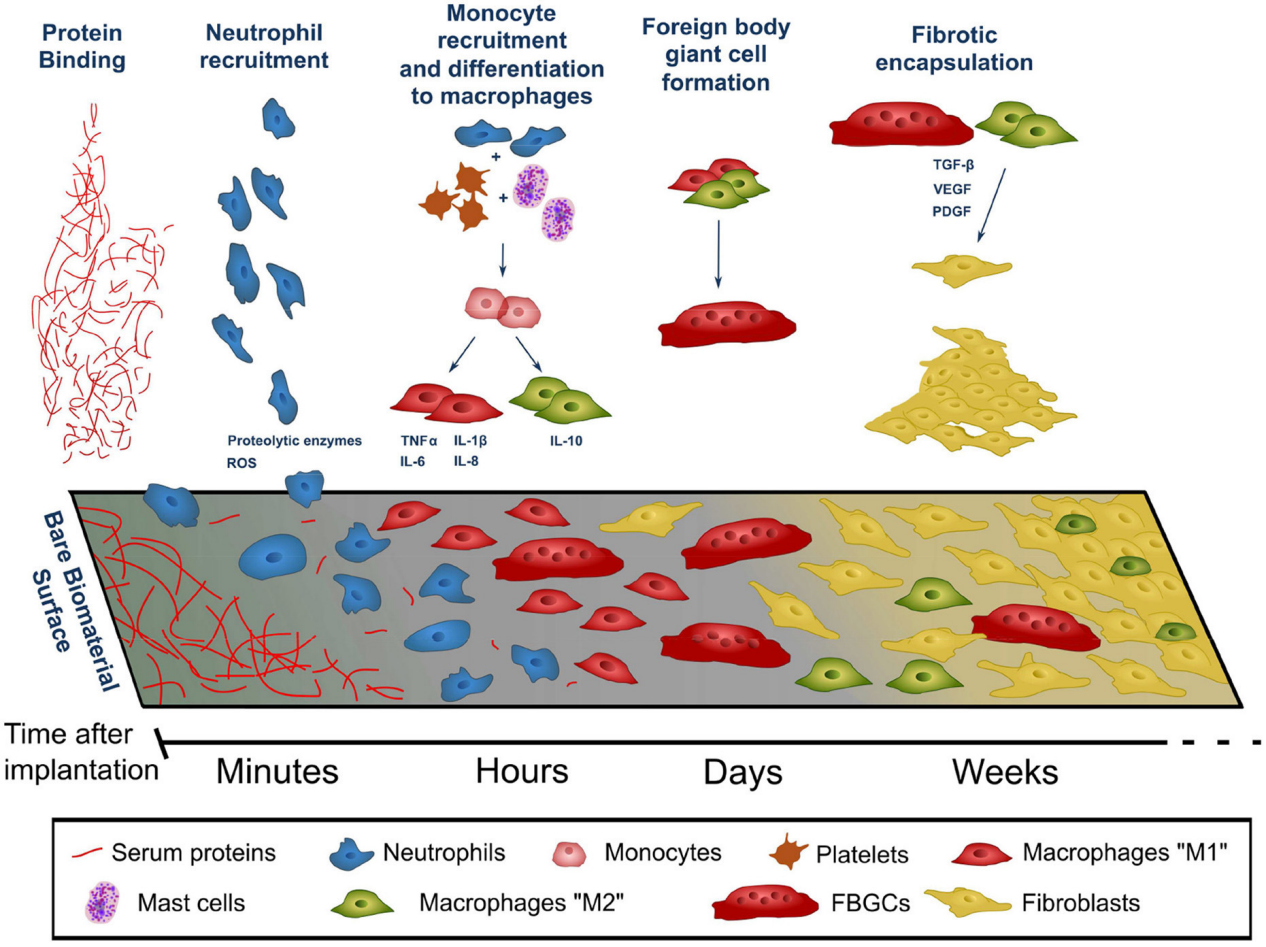
Timeline of the events leading to the development of the foreign body reaction to a material following its implantation into the body. The composition of the cell population adhered to the surface of the implant evolves over time following the initial implantation. Factors released by cells (indicated by blue text) contribute to the recruitment of further cells and progression of FBR. ROS, reactive oxygen species.

**Figure 2:**
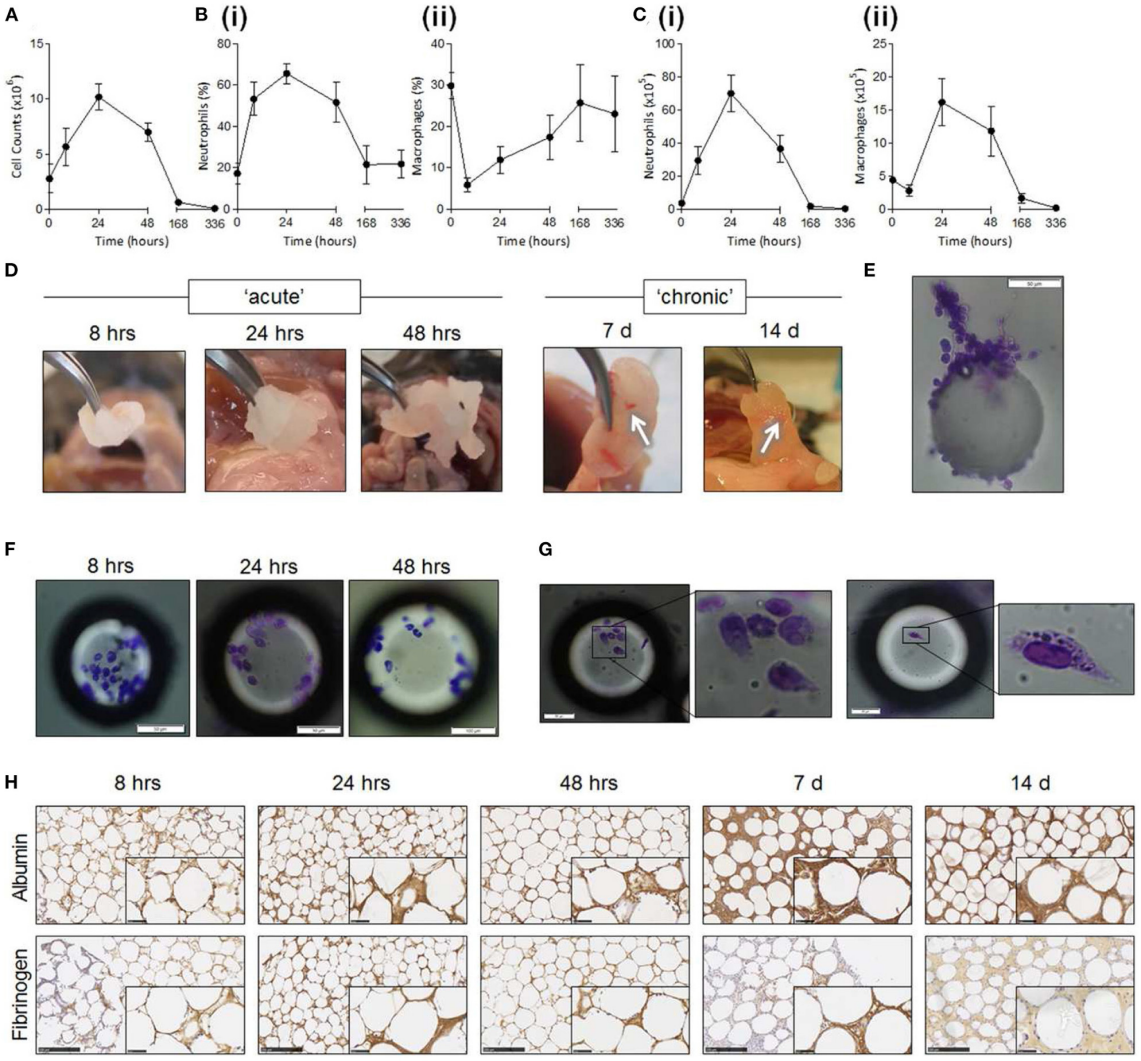
Foreign body reaction against intraperitoneally-implanted PMMA beads (125–180 μm). Reproduced under the terms of the Creative Commons Attribution License (Christo et al., 2016). Progression from acute to chronic FBR stages can be observed in counts of cells surrounding implants **(A-C)** and in the thickening of tissue capsule around them **(H)**. **(A–C)** Quantification of cell numbers in the peritoneum following bead implantation, of all cells **(A)**, neutrophils **(B,C)** (i), macrophages **(B,C)** (ii). **(D)** Explanted PMMA beads. Fibrotic encapsulation of beads visible at longer time points (white arrows). **(E–G)** Individual explanted beads stained for adhered leukocytes using Diff-Quik staining. **(H)** Bead aggregates sectioned and stained via immunohistochemistry for albumin and fibrinogen (brown staining). Scale bar: 250 μm.

Foreign body reaction (FBR) is an unavoidable process which takes place whenever any material becomes implanted into the body. The process of implantation injures the tissue around the foreign object, which triggers an inflammatory process. Over a period of weeks to months this inflammatory process develops into a fibrotic response, which envelops and isolates the implanted material. When the foreign material is implanted with the aim of delivering a therapy, both the acute (predominantly inflammatory) and chronic (fibrotic) stages of FBR pose significant challenges to its integrity and therapeutic function. The complex timeline of FBR is summarized in Figure 1, and exemplified in Figure 2.

### Acute FBR

The acute phase of FBR begins immediately after implantation. The tissue damage and extravasation of blood which inevitably occurs during the implantation process triggers an immediate rush of inflammatory-mediating cells to the area. Within seconds of implantation, proteins—many of which are derived from extravasated blood, such as albumin and fibrinogen—become non-specifically adsorbed to the surface of the implant (Figure 1—protein binding). This layer of proteins becomes a provisional matrix, through which cells gathering in the area can identify and interact with the foreign body. As time progresses, proteins of this provisional matrix undergo a dynamic process of adsorption-desorption, through which the smaller proteins which are initially found around the implant surface (such as albumin) are progressively replaced by larger ones. This process—known as the Vroman effect—can vary in protein composition and time course for different implanted materials, leading to differences in FBR across different materials even at this early stage (Wilson et al., 2005; Xu and Siedlecki, 2007).

The implantation process marks the beginning of a cascade of cellular events that make up FBR. Within minutes of implantation, neutrophils—the early responders in any type of tissue injury—migrate into the area. Neutrophils adhere to the protein layer surrounding the implant and begin to release factors which promote the progression of the inflammatory process (such as reactive oxygen species and proteolytic enzymes, Figure 1—neutrophil recruitment, Figure 2). Together with similar chemical signals resulting from blood clotting and mast cell activation, these factors increase vascular permeability and attract monocytes into the site of implantation. Once arrived, monocytes begin to differentiate into macrophages, which in turn proliferate and populate the lesion (Figure 1–monocyte recruitment and differentiation to macrophages, Figure 2). Within two days of implantation the initial wave of neutrophils has completely disappeared to give way to a population of macrophages (Anderson et al., 2008; Franz et al., 2011), which self-sustains itself by continuously proliferating and releasing chemoattractants that recruit further macrophages. These macrophages also mediate the core of the inflammatory response, releasing pro-inflammatory actors such as TNFα (tumor necrosis factor α), and interleukins IL-1b, IL-6, and IL-8 (Jones et al., 2007; Mesure et al., 2010). Up to this stage the inflammatory response to device implantation is very similar to that occurring in any injury—neutrophils rush to the area and recruit macrophages, which first eliminate invading threats and then mediate tissue repair. However, as macrophages populate the site of implantation, this initial acute inflammatory response develops into FBR.

As macrophages populate the lesion site they begin to adhere to and cover the exposed surface of the implant. This adhesion process is thought to be a critical component of FBR initiation, and occurs through integrins—a broad class of transmembrane proteins which bind to other proteins of the tissue environment—that specifically bind to the proteins adsorbed to the implant's surface. In particular, αMβ2 integrin is considered crucial for this initial adhesion stage as it specifically binds to serum proteins of the implant surface such as fibronectin and fibrinogen (Anderson et al., 2008). Following this initial adhesion stage macrophages undergo cytoskeletal remodeling. Bound macrophages flatten over the surface of the implant in an attempt to engulf and phagocytose it, and extend podosomes—structures specialized in proteolysis and extracellular matrix remodeling. Bound and activated macrophages also secrete chemoattractive factors which continue to recruit macrophages even after the initial implantation injury has resolved (Crowe et al., 2000).

The macrophage layer forming around the implant creates a defined and isolated space. Being unable to phagocytose the entire implant due to its large size, macrophages instead secrete factors into this space in an attempt to break down the foreign body and instead phagocytose the resulting fragments. The factors released by macrophages as a result of this frustrated phagocytosis include degrading enzymes and reactive oxygen species (Hansen and Mossman, 1987; Anderson, 2001). This macrophage attack poses a significant challenge to implant stability. Otherwise stable biomaterials can exhibit surface degradation and cracking when exposed to the hostile inflammatory environment created by macrophages (Labow et al., 2001; Wiggins et al., 2001), which may cause a breakdown of the implant or the leaching of toxic species into the tissue from the underlying layers.

If macrophages successfully degrade and phagocytose the implant during this acute phase of FBR, the reaction ends, and the tissue slowly returns to normal. Some implantable devices are designed to exploit this inflammatory process to degrade after they have carried out their therapeutic role. A common example of this are regeneration conduits: implants inserted into lesioned tissue designed to guide tissue regrowth and be degraded as the tissue heals (Arslantunali et al., 2014). In many cases, however, implants are required to remain implanted indefinitely to continue carrying out their therapeutic role. When in these cases macrophages fail to degrade the implant, FBR transitions into its chronic stage.

### Chronic FBR

The chronic phase of FBR is characterized by a transition from solely inflammatory to a fibrotic process. In contrast to the direct challenges to an implant's survivability posed by acute FBR, the chronic stage of FBR involves the encapsulation of the implant in a layer of fibrous tissue. This fibrotic layer acts as a barrier between the implant and the host tissue into which it was implanted, and can greatly difficult the delivery of any therapy into the surrounding tissue. FBR progressively transitions into its chronic fibrotic stage over the course of weeks after implantation and, unless the implant is destroyed or removed, it remains active indefinitely.

During the transition to the chronic stage of FBR macrophages switch from a pro-inflammatory activation phenotype (M1 macrophages) to an anti-inflammatory and tissue generation phenotype (M2 macrophages) (Mantovani et al., 2004; Lawrence and Natoli, 2011; Sridharan et al., 2015). This phenotypic transition is often observed as part of the normal wound healing process, marking the switch from the elimination of foreign threats in an injury to the healing of the tissue (Brown et al., 2012; Hesketh et al., 2017). During FBR, however, M2 macrophages instead play a role in the formation of a fibroblast and ECM-rich capsule that covers and isolates the implant.

M2 macrophages become central orchestrators of fibrosis during FBR, attracting and organizing fibroblasts to the implant's surface. M2 macrophages decrease inflammatory activity by releasing anti-inflammatory cytokines such as IL-10 (Klopfleisch, 2016). TGF-β released by these macrophages attracts local fibroblast populations, and induces their activation on arrival (Ignotz and Massagué, 1986). These activated fibroblasts adhere to the implant's surface and begin depositing layers of extracellular matrix proteins (Figure 1—fibrotic encapsulation). The activation of fibroblasts involves their transdifferentiation into myofibroblasts—a cell type also responsible for the formation of scar tissue and commonly found in wound healing (Hinz et al., 2007). PDGF—also released by macrophages—induces the proliferation of myofibroblasts, which over time cover the entire surface of the implant (Bonner, 2004).

A hallmark of FBR is also the fusion of macrophages into polynucleate foreign body giant cells (FBGCs) on the surface of the implant. Within days of implantation macrophages adhered to the implant begin to fuse with each other into much larger cells (Figure 1—foreign body giant cell formation), capable of phagocytosing larger particles (> 10 μm, in contrast to the < 5 μm that macrophages can phagocytose) (Anderson et al., 2008). The dynamics of the fusion process of macrophages into FBGCs appears to be tightly tied to the composition of the layer of proteins adsorbed to the implants surface, and therefore to the properties of the material itself (McNally and Anderson, 2002). While capable of phagocytosing larger particles than their non-fused counterparts, FBGCs release similar degradative and chemoattractive factors, and do not appear to play a unique role in FBR. Their striking morphology, almost exclusive to FBR, is however a useful tool for the identification of this reaction in tissue samples in both research and clinic.

The combination of macrophages, fibroblasts, and laid down extracellular matrix build up to create and entirely new tissue—the FBR capsule. As the capsule develops into a new tissue compartment new blood vessels have to extend into it in order to supply it with nutrients. VEGF and PDGF, released by the capsule tissue as it becomes anoxic, act as pro-angiogenic factors drawing in newly-forming blood vessels (Luttikhuizen et al., 2006). Further cell proliferation and ECM deposition allow the capsule to progressively thicken over months, until the implant becomes isolated from the surrounding tissue (Figure 2). The capsule eventually reaches a steady-state where macrophage activity is no longer intense enough to continue driving fibrosis further away from the implant. The thickness of the capsule and the time needed to reach this point varies greatly, and depends on multiple factors such as implant materials, size of implant, and location in the body. However, the capsule remains a living tissue—since the source of FBR remains present the capsule is capable of regrowing if damaged or removed, and can adapt if the properties of the enclosed implant change.

It is worth noting that the described process of FBR can differ in certain tissues. The most notable example is the central nervous system. Due to the tight control the body exerts over cell and molecule crossing from the bloodstream into the central nervous system, FBR here is driven by different cell types. Namely, inflammation is mediated by CNS-resident microglia (rather than macrophages), while fibrosis is driven by astrocytes instead of fibroblasts (Polikov et al., 2005; Salatino et al., 2017). Despite these differences, the general principles underlying FBR and the consequences (formation of a fibrotic capsule, inflammatory damage to the surrounding tissue) still apply (Polikov et al., 2005; Salatino et al., 2017). Despite the similarities in tissue composition and function that the central and peripheral nervous systems share, FBR in peripheral nerves is not driven by microglia and astrocytes, but by macrophages and fibroblasts like in most other tissues.

The FBR capsule poses a significant challenge to the therapeutic function of implantable devices. While the chronic stage of FBR involves a less aggressive immune attack to the implanted materials, the growing fibrotic capsule forms a physical barrier that interferes with the delivery of agents or sensing of signals in the surrounding tissue. While certain classes of implants—such as those carrying out a structural role, or delivering large amounts of therapeutic agents to large volumes of tissue—are less affected by the FBR capsule, its presence is an increasingly relevant problem. As implantable technologies continue to progress toward more refined devices capable of interacting with ever smaller subsets of tissue, the disruption of the tissue-implant interface by FBR becomes a leading cause of failure (Salatino et al., 2017; Spearman et al., 2018). Moreover, as this failure is likely to occur at chronic implantation timepoints (months), identification of the problem and optimisation of implant design at preclinical testing stages is challenging. Understanding FBR and implementing design choices that account for it therefore becomes a crucial step in the development of medical implants.

While the cellular processes of FBR are well characterized the properties of an implantable material that initially tag it as foreign are not understood. As a result, FBR to implants cannot be entirely avoided. Instead, a successful implant capable of carrying out its therapeutic function for its intended lifetime needs to accommodate some degree of FBR, and implement design choices to ensure that this severity is not exceeded.

### Implant Design Considerations for FBR

FBR is fundamentally tied to tissue trauma (Wang et al., 2015; Klopfleisch and Jung, 2017). Not only is the initial implantation of a device the trigger that begins the cellular events leading to FBR, but subsequent trauma around the implanted device leads to further inflammation and worsens ongoing FBR. Consequently, one of the most effective strategies to reduce FBR is designing implants that minimize tissue trauma.

**Figure 3:**
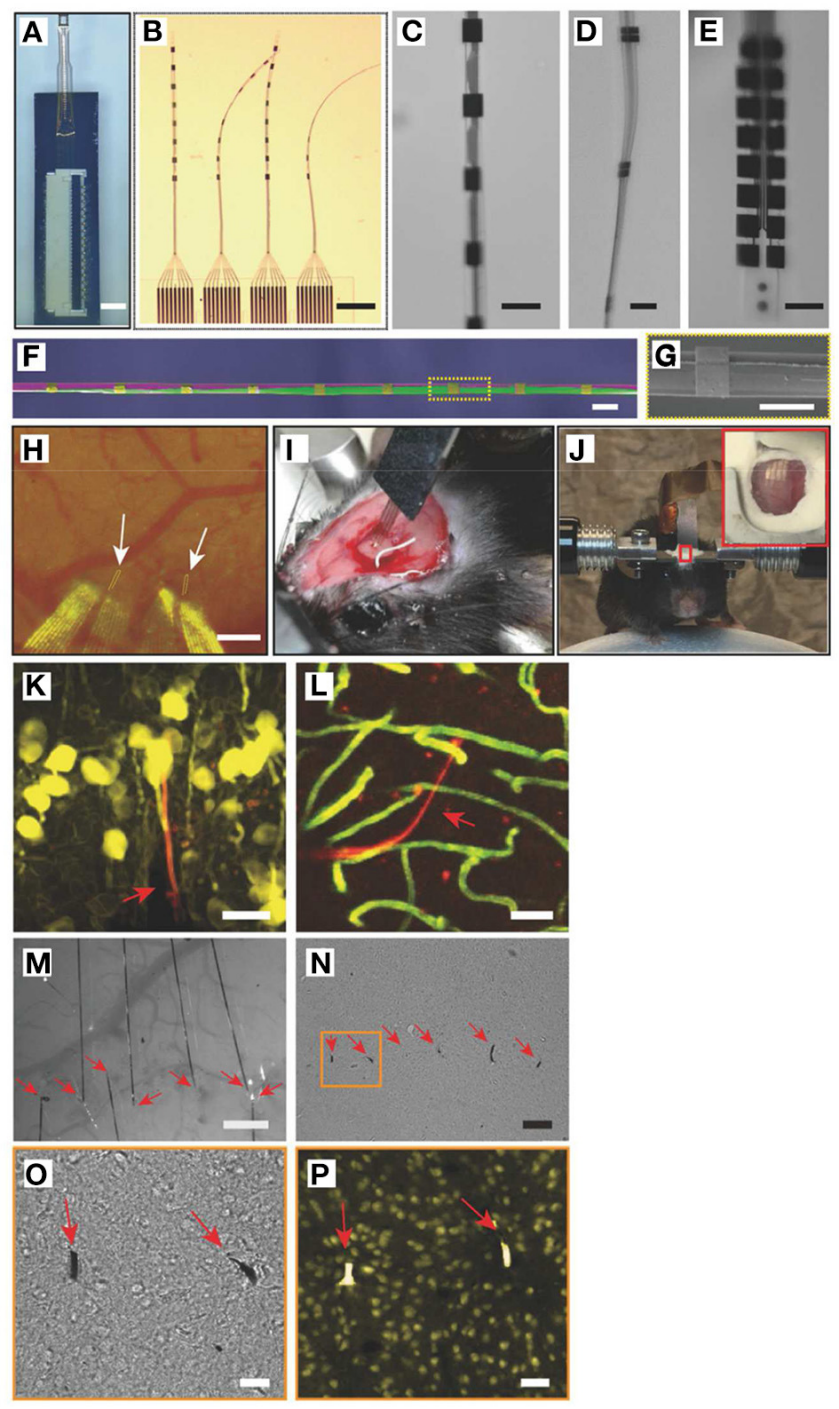
Implantation of flexible neural probes using stiff shuttles to minimize trauma and FBR. Reproduced under the terms of the Creative Commons Attribution License (Wei et al., 2018). **(A–G)** Flexible NET-e devices containing multiple electrodes. Hole to connect with the shuttle tool seen at the bottom of **(E)**. **(H–J)** Implantation of NET-e devices into the brain using a custom designed and fabricated carbon fiber shuttle device. Entry site indicated by white arrows. **(K–P)** Tissue response following long-term (2 month: **K–L**; 4 month: **M–P**) implantation of NET-e devices. No significant neuronal death or vasculature disruption seen. Neuronal staining in yellow (NeuN, **K,P**). Vascular staining in green **(L)**. Devices seen in red fluorescence or pointed by red arrows. Scale bars 50 μm **(K–N)**, 10 μm **(O,P)**.

Implants begin interacting with the body at the moment they undergo insertion into tissue. While implant design is often tailored to the location in the body which it will inhabit for its therapeutic lifetime, it is important to remember that delivering the implant to that location is a key step with consequences for the device's performance. Tissue damage during implantation is responsible for the initial inflammatory wave attracting cells to the area, and the resulting extravasated blood proteins provide the first interface for the cells to interact with the implant. It is therefore unsurprising that the degree of implantation trauma is linked to the degree of FBR (Wang et al., 2015). While implantation trauma cannot be totally eliminated, implants can be designed accounting for the surgical implantation procedure. For example, some brain probe designs incorporate biodegradable stiff material shanks that enable easy penetration into tissue, degrading thereafter and leaving behind the softer probe (Kozai et al., 2014). Such designs simplify the implantation procedure, eliminating the need for bulkier, more damaging tools which may otherwise be necessary to deliver the probe, and are associated with a lower amount of tissue damage (Kozai et al., 2014). Alternatively, flexible implants can be designed to be combined with purposedly-designed shuttle tools for low trauma implantation into tissue (Figure 3). Implants that take into consideration the mechanics of the implantation procedure and ease of surgical handling can minimize implantation trauma and FBR. Strategies not directly related to the implant itself can also aid in reducing implantation trauma, such as targeting of more easily accessible tissues. Imaging-guided surgical systems can be used to avoid major blood vessels and reduce blood extravasation (Spetzger et al., 1995), optimizing the implantation procedure.

Once implanted, devices continue to interact with the surrounding tissue—often in damaging ways. Tissue is subject to continuous motion at various rates and length scales, due to the host's breathing, blood pumping, movement, and locomotion. While implanted devices will also experience these movements, differences in mechanical properties between implant and surrounding tissue will lead to a mismatch in how the two move and deform in response. As implants fail to move together with tissue, the compression and sliding along the interface causes damage to the surrounding tissue (Goldstein and Salcman, 1973; Sharp et al., 2009; Barrese et al., 2016). This not only leads to local inflammation—therefore further exacerbating FBR—but can also permanently destroy the surrounding target tissue which may be the therapeutic target of the implant. This mechanical mismatch is particularly relevant for implants containing electronic components, as silicon-based components are orders of magnitude stiffer than biological tissues. In order to minimize FBR, implants have begun to experience a shift toward soft or flexible designs, capable of mechanically coupling with their local tissue environment. Particularly in neuroprostheses implanted in nervous tissue—some of the softest tissue in the body—implants implementing flexible or stretchable materials can achieve a much lower degree of FBR and tissue damage when chronically implanted (Nguyen et al., 2014; Minev et al., 2015; Lacour et al., 2016; Capogrosso et al., 2018). Smoother implant designs, avoiding sharp corners which can be particularly damaging to tissue when motion between the two occurs, can also be implemented to decrease tissue damage and chronic FBR (Veiseh et al., 2015).

Material chemistry is also tightly linked to the body response and FBR. Implantable materials need to be carefully chosen to not contain any toxic species which may damage host tissue or promote inflammation—a feature generally englobed under the term “biocompatible” (Williams, 2008). This not only includes material chemistry at the surface of the implant, but also components which may leach from within overtime or become exposed to the body if surface cracking occurs. Over decades of work, the field of implantable material research has selected materials that exhibit good biocompatibility, including polymers (e.g., silicones, polyethylene, polyimide), metals (e.g., platinum, gold) and ceramics. Certain features of material chemistry can also be exploited to beneficially influence FBR, such as the use of low-fouling materials which prevent FBR-triggering non-specific protein adsorption (Figure 1) (Zhang et al., 2013; Xie et al., 2018). A deeper discussion of the interactions between material chemistry and FBR, and of the choices of materials in different implants, are beyond the scope of this review and are discussed elsewhere (Williams, 2008; Hassler et al., 2011; Zarrintaj et al., 2018; Mariani et al., 2019).

In recent decades, more active steps have begun to be taken to directly tackle FBR. Active strategies meant to directly interfere with the cellular events leading to FBR have been implemented in implantable devices since the incorporation of anti-inflammatory compounds in pacemaker leads in the late twentieth century (Mond et al., 2014). Inflammation, however, plays an active role in other biological processes such as repair of tissue damaged around the implantation site, making the use of anti-inflammatory compounds not always possible. In recent years, as the finer molecular network underlying FBR are becoming better understood, new and more specific targets to interfere with FBR have become available. This new generation of active FBR treatments (contrasting with the passive strategies relating to implant design discussed above) are discussed later in this review.

## Nerve Neuroprosthetics and FBR

Neuroprosthetic implantable devices deliver a therapy to a patient by interfacing and interacting with their nervous tissue. This interface is typically electrical in nature—electrodes in the implant record the action potential activity in the nearby neuronal population, and/or activate nearby neurons through electrical stimulation. While potentially able to address a myriad of conditions, neuroprosthetics are a class of implantable device particularly vulnerable to FBR. The fibrotic capsule forming around the implant not only damages and displaces the target neuronal tissue, but also forms a high-impedance layer that dampens the weak electrical signals produced by neurons and dissipates stimulating electrode currents. FBR is currently the key culprit behind chronically-implanted neuroprosthetic implant failure (Polikov et al., 2005; Salatino et al., 2017), and poses a major hurdle to the clinical translation of new neuroprosthetic technologies.

Neuroprosthetics have frequently been targeted to the central nervous system, for purposes such as the treatment of Parkinson's disease and tremor symptoms (Anderson and Lenz, 2006), restoration of hearing (Colletti et al., 2009), or treatment of epilepsy (Sprengers et al., 2017). The peripheral nervous system, however, is becoming an increasingly attractive target for the delivery of therapies to restore lost or abnormal function. Nerves form a fine network of pathways through which the brain communicates with all structures around the body. By targeting the correct nerve, an implant can activate, modulate, or monitor a specific body structure—such as organs (e.g., bladder, pancreas), muscles (inducing movement), or skin (tactile sensation).

Despite its great therapeutic potential, the peripheral nervous system is particularly vulnerable to FBR. Unlike the brain and spinal cord, which are enclosed in protective bony structure, nerves run between muscles and organs. Body movements, as caused by normal activities such as locomotion or limb movements, subject nerves to constant tensile and compressive forces. When an implant is present, these mechanical challenges can lead to nerve trauma, exacerbating FBR—particularly when stiff implant materials, contrasting with the very soft nerve tissue, are present (Lacour et al., 2016). Additionally, while the CNS is considered an immune privileged site (Louveau et al., 2015) where inflammation and fibrosis are more subdued than elsewhere in the body, the peripheral nervous system develops the typical severity of FBR (Spearman et al., 2018).

**Figure 4:**
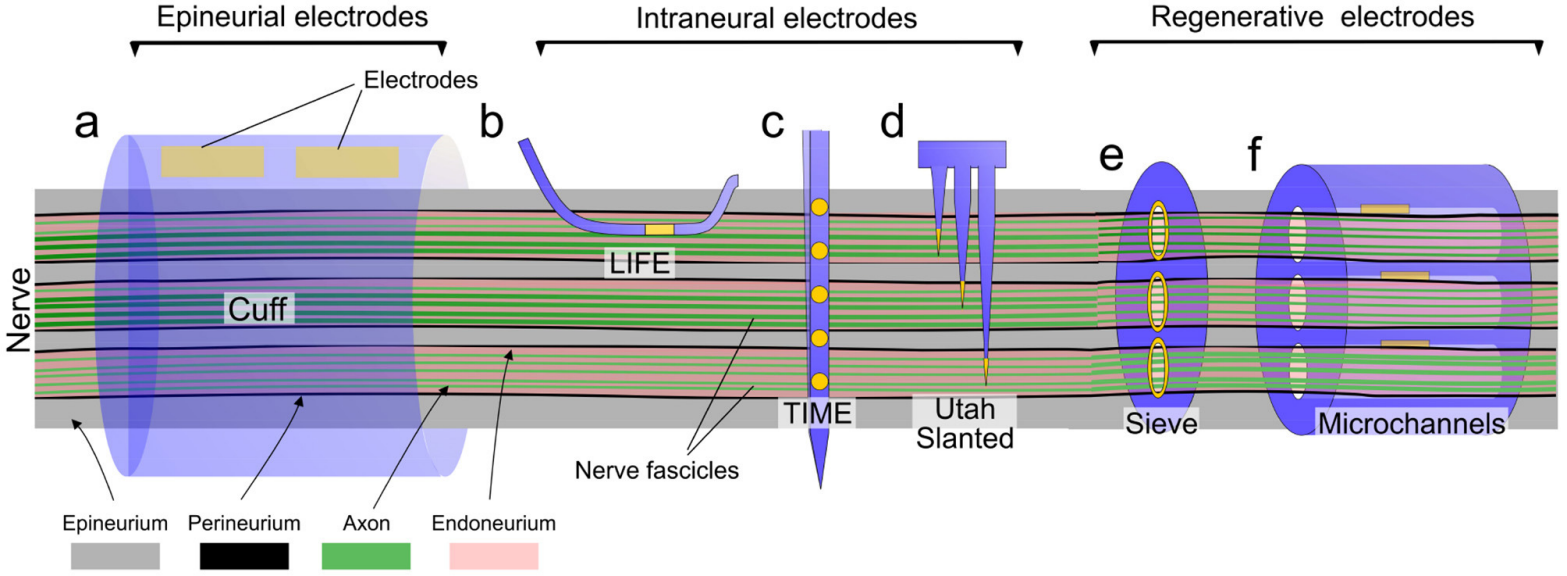
Overview of different types of nerve neuroprosthetic designs and their implantation locations within the nerve anatomy. **(a)** Epineurial cuff electrodes, **(b)** Longitudinally implanted intrafascicular electrodes (LIFE), **(c)** Transverse intrafascicular multichannel electrodes (TIME), **(d)** Utah slanted electrode array, **(e)** Regenerative sieve electrodes, **(f)** Regenerative microchannel electrodes.

Nerve neuroprosthetic technologies are very varied, with different designs eliciting different kinds of responses in tissue (Table 1). The degree of nerve invasiveness is often used as a metric to group together different classes of neuroprosthetics. Nerves are complex structures formed by three main types of tissue. The nerve endoneurium houses the axons of neurons, projecting throughout the entire length of the nerve and transmitting signals across the body in the form of action potentials. The endoneurium is bundled by a tightly packed layer of cells, making the nerve perineurium. While some nerves contain a single of these endoneurial bundles, others contain multiple distinct bundles which are individually referred to as nerve fascicles. Finally, the entire nerve is wrapped in a layer of ECM-rich tissue—the epineurium—which provides structural support and mechanical protection to the nerve (Figure 4).

**Table 1:** Summary and comparison of the nerve response and FBR to various classes of nerve neuroprosthetics.

**Nerve neuroprosthetic type**	**Implant material**	**Implantation period**	**Nerve tissue and FBR response**	**Fibrotic area/thickness**	**Animal model**	**References**
Cuff	Silicone	133–137 days	Nerve structure reorganized to match cylindrical cuff. FBR capsule developed around inner edge of cuff. Collagen-rich tissue growth between FBR capsule and nerve fascicles.	2.13–2.65 mm^2^	Cat	Romero et al., 2001
Spiral cuff	Silicone	28–34 weeks	Nerve morphology deformed by presence of cuff. Abundance of abnormally thinly-myelinated axons. Thinning of the perineurium. Extensive fibrosis in epineurium. FBR capsule around the inside of cuff.	n.m.	Cat	Grill and Mortimer, 2000
Spiral cuff	Silicone	18 hours, 7 days, 1 month	Reshaping of nerve morphology seen as soon as 18 hours. Fibrotic capsule seen from 7 day time point. Upregulation of inflammatory markers at 18 hours and 7 days, which decreased by 1 month.	n.m.	Rat	Vince et al., 2005
Cuff	Hydrogel or PET	6 weeks, 2 months	Soft (hydrogel) cuffs led to no inflammation or any perceivable fibrotic thickening of the nerve epineurium. Stiff (PET) cuffs developed extensive nerve inflammation and fibrosis, and some loss of axons.	n.m.	Rat	Liu et al., 2019, 2020
FINE cuff	Silicone	3 months	FINE cuffs lead to a fibrotic thickening of the epineurium, without axonal damage as long as only moderate pressure is applied.	4.33–4.36 mm^2^	Cat	Leventhal et al., 2006
LIFE	Polyimide	3 months	No substantial nerve or axon damage. Focal but chronic inflammation and scar formation around implant.	0.04 mm^2^	Rat	Lago et al., 2007
TIME	Polyimide	2 months	No significant loss of axons or nerve activity. Thin fibrotic capsule seen surrounding the implant intraneurally.	n.m.	Rat	Badia et al., 2011b
LIFE	Parylene C	Up to 32 weeks	Inflammation (macrophage presence) increased in the nerve following implantation, reaching a maximum at 2 weeks post-implantation. A fibrotic capsule developed surrounding the implant and thickening over time.	~50 μm by 32 weeks	Rat	de la Oliva et al., 2018
SELINE	Polyimide	Up to 165 days	Inflammation (macrophage presence) and fibrosis increased in the weeks after implantation, but decreased by 165 days post-implantation. Loss of axons close to the implant at chronic time points.	~0.07 mm^2^ at 28 days, ~0.04 mm^2^ at 165 days	Rat	Wurth et al., 2017
Slanted Utah array	Silicon	Up to 350 days	Axonal degeneration in fascicles penetrated by the array shanks. Extensive inflammation surrounding the shanks, particularly close to the base.	n.m.	Cat	Christensen et al., 2014
Sieve	Polyimide	2 to 12 months	Nerve fiber regeneration took place between 2 to 6 months post-implantation. Axon numbers declined (axonal death) 6 to 12 months post-implantation.	n.m.	Rat	Lago et al., 2005
Microchannel	Silicone	3, 6, and 12 months	Axon regeneration through microchannels was robust in the first 3 months. This was followed by a decrease in axon number as the thickness of the fibrotic capsule around the inner channel wall increased.	34 μm	Rat	FitzGerald, 2016

*Studies that did not report on the response of tissue following implantation have not been included. N.m., FBR capsule thickness not measured in study; PET, Polyethylene terephthalate; LIFE, longitudinally implanted intrafascicular electrodes; TIME, transverse intrafascicular multichannel electrodes; FINE, flat interface nerve electrode; SELINE, self-opening neural interface*.

**Figure 5:**
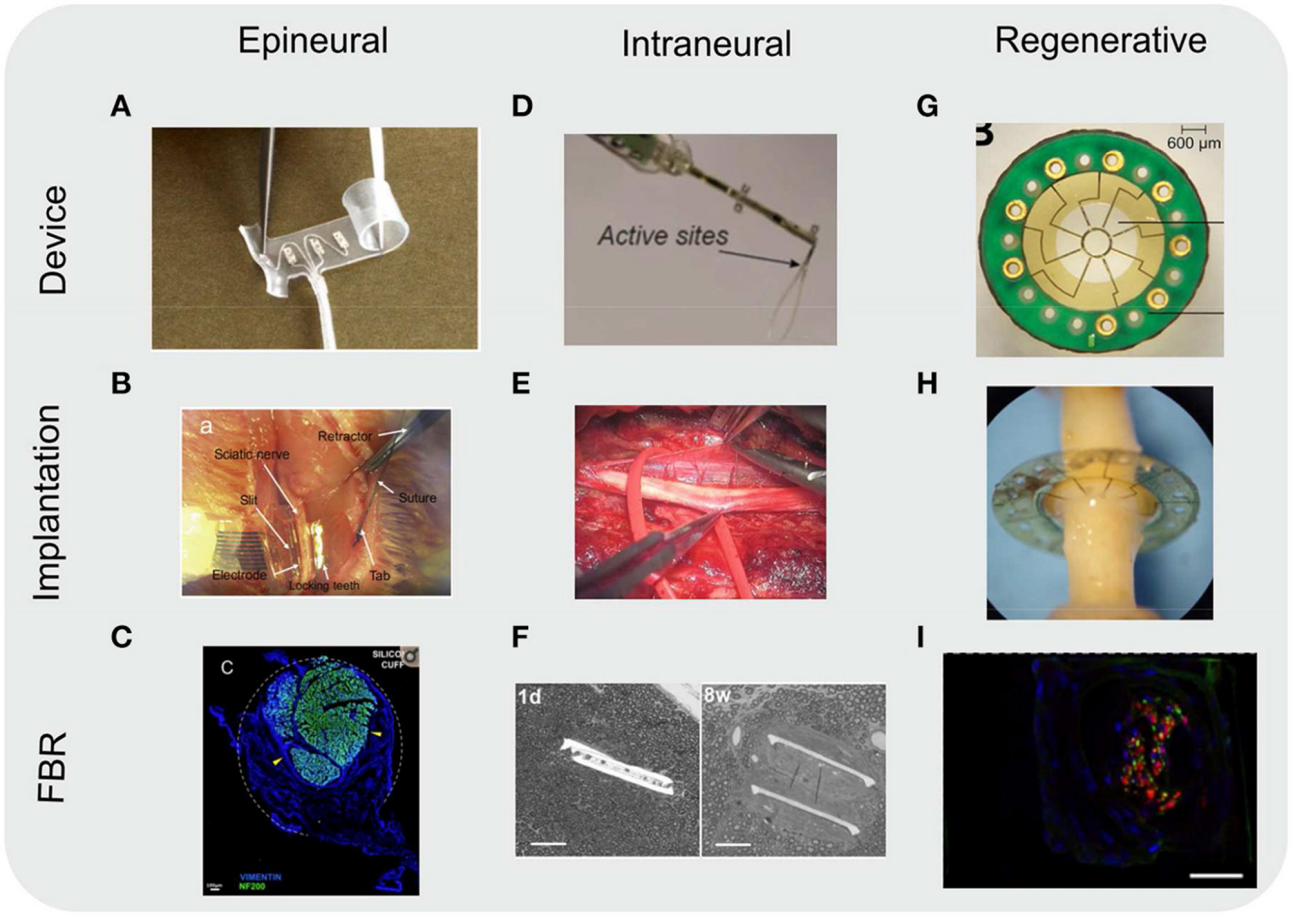
Images of various types of nerve neuroprosthetics. Images included of devices as fabricated (device), implanted into rodent sciatic nerves (implantation), and of the resulting FBR in tissue (FBR). **(A-C)** Epineurial cuff electrodes. **(C)** Cross-section of a rat sciatic nerve implanted with a silicone cuff 30 days post-implantation. Nerve (represented by immunoflurescently labeled axon marker NF200, green) take up a small portion of the cuff, with the rest filled with fibroblasts (vimentin, blue) due to FBR to the cuff. Dotted white line indicates approximate position of the cuff. Yellow arrowheads indicate compression of the nerve due to growing fibrotic tissue. Scale bar: 100 μm. **(D–F)** Intraneural electrodes, either transverse intrafascicular multichannel electrodes (TIME) **(D,E)** or longitudinally implanted intrafascicular electrodes (LIFE) **(F)**. **(F)** Toluidine blue-stained images of nerve cross-sections. The two branches of the LIFE parylene C implant (slits in tissue) are found in close proximity to the axons (small circular structures) 1 day (1 d) post-implantation. By 8 weeks post-implantation (8 w) a fibrotic capsule (seen as a smooth ring around the implant slits) develops, displacing the axons. Scale bar: 50 μm. **(G–I)** Regenerative sieve **(G,H)** and microchannel **(I)** electrodes. **(I)** Cross-section of a rat sciatic nerve regenerated through a PDMS microchannel implant (image of a single microchannel) 12 weeks post-implantation. Nerve tissue (represented by immunoflurescently labeled axon marker NF, green; and Schwann cell S100, red) take up a small portion of the microchannel cross-section. The remainder of the microchannel is filled with other cell types (nuclear stain DAPI, blue), likely macrophages and fibroblasts as a result of FBR to the microchannel walls. The outline of the image represents the approximate position of the microchannel walls. Scale bar: 20 μm. All images reproduced under the terms of the Creative Commons Attribution License: **(A)** (Christie et al., 2017), **(B)** (Elyahoodayan et al., 2020), **(C)** (González-González et al., 2018), **(D)** (Zelechowski et al., 2020), **(E)** (Strauss et al., 2019), **(F)** (de la Oliva et al., 2018), **(G,H)** (MacEwan et al., 2016), **(I)** (Musick et al., 2015).

Due to this structure, more invasive implant designs with electrodes positioned close to axons in the endoneurium can achieve better electrical recording/stimulation, and may selectively target specific subpopulations of axons (e.g., individual fascicles within the same nerve). More invasive implants, however, can lead to trauma and FBR closer to the fragile nerve axons, potentially leading to the long-term failure of the implant (Figure 5). This invasiveness-selectivity trade-off is a key factor influencing the design of nerve neuroprosthetic implants.

Based on the degree of invasiveness nerve neuroprosthetic designs can be categorized into three different groups (summarized in Figures 4, 5). Epineurial cuff electrodes are the least invasive, placed around the outside of the nerve without breaching the epineurium. Intraneural penetrating electrodes locally breach the epineurium and perineurium of the nerve, piercing into the endoneurial compartment. Finally, the most invasive group are regenerative nerve electrodes, which exploit the regenerative properties of the peripheral nervous system in order to remain embedded in regrowing nerve tissue following an injury. While there is a wide range of nerve implant designs covering the entire invasiveness-selectivity spectrum, these subdivisions provide a useful and commonly used way of categorizing implants (Navarro et al., 2005) and discuss their implications in the context of FBR.

### Epineurial Cuff Electrodes

Cuff electrodes are the simplest type of neuroprosthetic design. Cuff implants typically consist of a hollow cylinder of insulating material containing one or more pair of electrodes along its inner surface, which is wrapped around the outermost layer of the nerve (the epineurium) during surgical implantation. Their low invasiveness, simple design and simpler implantation procedure has made them arguably the most successful type of nerve neuroprosthesis in reaching the human clinic. Their low invasiveness makes them ineffective at selectively recording electrical signals from subsets of axons within a nerve, as the connective tissue of the epineurium dissipates the low amplitude axon action potentials. Cuff electrodes are, however, effective at providing a therapeutic effect through whole nerve electrical stimulation.

Nerve stimulation carried out through implantable nerve cuffs is currently used at the clinical level to treat a variety of conditions. These include sacral nerve stimulation to restore bladder and bowel function (Brindley et al., 1982; Johnston et al., 2005) and peroneal nerve stimulation for the treatment of foot drop (Liberson et al., 1961; Burridge et al., 2007). Electrical stimulation of the vagus nerve—a cranial nerve connecting the brain with many of the body's organs—is also becoming an increasingly popular choice for the treatment of conditions such as epilepsy (Uthman et al., 2004) and depression (Schachter, 2002; Nemeroff et al., 2006). Phrenic nerve stimulation, also known as diaphragm pacing, has also been recently introduced into clinical practice as an aid for ventilation in spinal cord injury patients (Hirschfeld et al., 2008).

Outside of the clinic, nerve recordings using cuff electrodes are also being explored as potential therapeutic avenues. Although more challenging to carry out compared to nerve stimulation due to the distance between electrodes and the weak electrical signals transmitted by axons, with an ECM-rich epineurium separating the two, motor and sensory nerve signals gathered using cuff electrodes have been used to guide and fine-tune prostheses such as artificial limbs in human patients (Micera et al., 2001; Navarro et al., 2005; Raspopovic et al., 2010). The comparatively large size of cuff electrodes and their ease of use have also made cuff electrodes a popular platform for the development of new neuroprosthetic-related technologies, such as wireless ultrasound-driven nerve stimulation (Piech et al., 2020), self-wrapping designs (Zhang et al., 2019), and self-healing, stretchable devices that accommodate for tissue growth as an animal grows (Liu et al., 2020.

Due to the position in which cuff electrodes are implanted, FBR to these cuffs develops predominantly in the epineurium of the host nerve. While this is useful to avoid damage to the fragile neuronal axons in the endoneurium as a consequence of inflammation, the epineurium already poses a significant barrier to communication between nerve and electrodes. A thickening of the epineurium as part of FBR can dissipate the already weak electrical signals transmitted by axons, limiting the use of these devices for electrical recording applications in long-term implantations (Navarro et al., 2005). This effect can be compounded when the cuff fits the nerve too loosely. Gaps between the nerve epineurium and implant are prone to filling with fibrotic tissue (Romero et al., 2001), and can increase trauma to the nerve due to mechanical shear damage during movement.

While tightly fit cuffs can offer better results, excessive nerve compression can severely impact long-term nerve health. Although the nerve connective tissue absorbs tensile forces along its axis, compressive forces around the nerve are transmitted to the fragile axons in the endoneurium. Nerve compression is associated with axonal death and inflammation (Krarup et al., 1989; Grill and Mortimer, 2000; Vince et al., 2005), which can in turn exacerbate FBR to the implant (Romero et al., 2001). Long-term nerve compression can also contribute to the development of neuropathic pain (Campbell and Meyer, 2006; Yalcin et al., 2014).

Flexible and stretchable materials such as PDMS have been an effective option to build the body of the cuff, able to gently squeeze around the nerve epineurium without excessive compression. From a chemical perspective, PDMS is well characterized to be inert and biocompatible, not releasing toxic species of exacerbating the inflammatory response and being well-suited for long-term implantation (Williams, 2008; Hassler et al., 2011). PDMS is now the material of choice for most nerve cuffs in clinical practice, including those for vagus nerve (Ben-Menachem et al., 2015) and sacral nerve stimulation (Brindley et al., 1982; Johnston et al., 2005). PDMS cuffs develop lower degrees of trauma and FBR than cuffs made from other flexible materials such as metallic meshes (Christensen and Tresco, 2018), matching the pattern seen in other low-stiffness materials (Liu et al., 2019, 2020). However, deformable substrates such as PDMS pose difficulties for the fabrication of electronics (Lacour et al., 2016). This becomes an increasingly relevant challenge as nerve neuroprosthetics evolve into more elaborate designs, aiming to achieve higher resolution communication with the host nerves.

Despite the sensitivity of nerves to poor-fitting nerve cuffs, certain cuff electrode designs purposely apply compressive forces to the nerve in order to achieve better interfacing. Specifically, the FINE (flat interface nerve electrode) is designed with a flattened, elliptical bore in order to flatten out large cylindrical nerves. Large sensorimotor nerves often contain axons bundled into separate fascicles each leading to a different muscle, which can each be targeted for better therapeutic selectivity. However, fascicles are typically packed tightly together in the nerve cross-section, and classical cuff designs struggle to selectively stimulate individual fascicles. FINE cuffs are designed to flatten the nerve, distributing its fascicles over a wider cross-section, making them accessible to sets of electrodes lining the inner surface of the cuff. This designed has been used to selectively restore sensory perception in human arm amputee patients (Tan et al., 2014). While excessive compressive forces are associated with axonal death and increased FBR, moderate nerve reshaping with FINE implants does not seem exert sufficient force to cause axon death or more connective tissue deposition than seen in wider nerve cuffs (Tyler and Durand, 2003; Leventhal et al., 2006).

Advances in fabrication technologies are also opening new possibilities for the development of better long-term performing cuff implants. Nerves can be scanned in reconstructed in three dimensions in order to later 3D print cuffs perfectly adapted to their anatomy (Johnson et al., 2015), potentially minimizing mechanical trauma and FBR at the nerve-implant interface. 3D printed implants can be compatible with electrode manufacturing techniques (Haghiashtiani and McAlpine, 2017), opening the door for future perfect-fit epineurial cuff electrodes suitable for clinical use.

### Intraneural Penetrating Electrodes

Intraneural electrodes are designed to penetrate the outer layers of the nerve and position the electrodes in closer proximity to the axons—often directly inside the endoneurial compartment. The need to penetrate through connective tissue layers in order to reach neuronal tissue places similar design needs to those of penetrating electrodes used in the brain, and indeed some intraneural nerve electrodes stem from designs used in brain applications. Penetrating electrodes are placed in closer proximity to axons and without the presence of electrically insulating connective tissue layers. Their location in the nerve anatomy allows them to carry out good quality electrical recordings, potentially being able to distinguish between signals from different groups of axons within one same nerve fascicle. Being embedded within the nerve tissue, penetrating electrodes are also better anchored to their target tissue which prevents slippage between the two and ensures that the same groups of axons can be monitored over time. While also useful for electrically stimulating small groups of axons within the nerve, the small size of electrodes and the manufacturing techniques used in penetrating devices make them less effective at delivering widespread current for whole nerve activation. For nerve stimulation at the clinical level, epineurial cuffs remain the preferred choice (Navarro et al., 2005).

Penetrating electrodes, however, face a disadvantage in the need to rupture through nerve compartments during implantation. This process not only generates more trauma than an epineurial device, but also localizes the trauma closer to axons. This can not only lead to the development of a fibrotic capsule deep within the nerve tissue, but can also lead to nearby axon death due to the inflammation associated with FBR. Moreover, by remaining implanted within the nerve tissue, shear damage due to mechanical mismatch between the two becomes a major source of further trauma that can potentially worsen axon death and fibrosis.

While intraneural penetrating electrode designs have not yet reached widespread use in the human clinic, a wide range of designs have been developed over the years in the research environment. Longitudinal-interfascicular electrodes (LIFE), consisting of a long flexible body with a single electrode implanted into the endoneurium of a nerve fascicle with the aid of a rigid needle, were one of the earliest penetrating designs developed (Malmstrom et al., 1998). More recently, updated LIFE designs manufactured using microfabrication technology and containing multiple electrodes along its length have also been developed (thin-film LIFE) (Navarro et al., 2007). LIFE implants are effective for long-term recording and stimulation applications, having for example been used in humans for the recording of volitional limb movement and to elicit skin sensation of touch/pressure (Dhillon et al., 2004, 2005; Micera et al., 2008).

While LIFE implants are limited to interfacing with a single nerve fascicle, transverse intrafascicular multichannel electrodes (TIMEs) are designed to penetrate through the entire nerve cross-section and establish connection with multiple nerve fascicles. By stimulating one or multiple fascicles simultaneously, TIMEs are able to produce a wider range of distinct nerve activation patterns. This can, for example, be used to reproduce a wider range of distinct sensory perceptions in amputee patients (Strauss et al., 2019). TIME implants are also fabricated from flexible materials such as polyimide, and are also inserted with the aid of a microneedle which is removed after surgical implantation (Boretius et al., 2010). TIMEs are able to both stimulate (Badia et al., 2011b) and record from Badia et al. (2016) separate nerve fascicles within nerves independently, and coupled with artificial hand prostheses have been used to restore sensory perception in human hand amputee patients (Raspopovic et al., 2014; D'Anna et al., 2019). Stimulating TIMEs have also been used to restore sensory perception in leg amputees, combined with pressure sensor-equipped leg prosthetics (Petrini et al., 2019).

LIFE and TIME nerve neuroprosthetics share a key similarity in their design: the use of flexible materials. Flexible implants are able to better deform with the nerve tissue, as the two move around together during normal body activity. Flexible implants in general show improved tissue compatibility compared to stiffer equivalents, minimizing trauma and FBR arising from shear damage (Lacour et al., 2016; Salatino et al., 2017). This is particularly relevant for soft and fragile tissues, or tissues which are constantly exposed to movement—two characteristics which apply to the peripheral nervous system. Though LIFE and TIME implants still induce a substantial amount of trauma during the implantation procedure, they elicit a limited degree of FBR. While a thin fibrotic capsule forms around these flexible devices over chronic implantation time points, the degree of fibrosis is mild enough to permit stable long-term recordings, with no widespread inflammation or axon damage seen (Lago et al., 2007; Badia et al., 2011a). The mechanical matching with tissue renders these implants very robust, exhibiting very little damage or delamination after implantation (Cvančara et al., 2020). Self-deploying anchors can also be incorporated into intraneural designs, minimizing movement and the associated trauma following implantation, as seen with self-opening neural interface (SELINE) devices (Cutrone et al., 2015), and generating minimal FBR (Wurth et al., 2017). Combined with potential improvements in the implantation procedure, flexible penetrating nerve implants such as these hold great potential for long-term therapeutic applications and translation into clinical use, as highlighted by the several recent uses of TIMEs in human patients (D'Anna et al., 2019; Petrini et al., 2019).

Most of the materials used in these flexible devices are chosen to have well-established biocompatibility—being chemically inert to the body and remaining so for long periods of implantation—while also being compatible with thin-film fabrication techniques. Polyimide is particularly popular for nervous system implant applications—including the PNS. Polyimide is biocompatible and exhibits low cytotoxicity *in vitro*, and remains stable for long periods of implantation (Richardson et al., 1993; Rubehn and Stieglitz, 2010; Hassler et al., 2011). Parylene C has recently arisen as a good material option for peripheral nerve devices (de la Oliva et al., 2018), and has a well-established history of biocompatibility and biostability (Chang et al., 2007; Winslow et al., 2010; Hassler et al., 2011).

Stiff material penetrating arrays have also seen widespread use as nerve neuroprosthetics. Utah arrays were developed in the early 1990s as a means to deploy large numbers of electrodes to deep layers of the brain (Normann and Fernandez, 2016), and have since been adapted for use in nerves (Branner and Normann, 2000; Branner et al., 2001). These implants consist of arrays of stiff silicon shanks with individual electrodes exposed at their tips. During implantation the shank array is pierced directly into the neural tissue. The variety fabricated for use in nerves has shanks of variable depth, allowing electrodes to be deployed into different fascicles and giving them the name of slanted Utah arrays. In contrast to other penetrating probes, Utah arrays incorporate up to 100 electrodes which can be deployed across the entire cross-section of the nerve—ensuring that the entire nerve axon population can be interfaced with. Slanted Utah arrays have been used for both stimulation (restoration of sensory perception) and recording (monitoring of motor activity) applications in human hand amputee patients (Clark et al., 2014; Davis et al., 2016; Wendelken et al., 2017). Nerve recordings with these implants were able to guide the movement of a virtual hand prosthesis over a period of 30 days, indicating that robust recordings can still be achieved during the onset of the chronic stages of FBR (Davis et al., 2016).

Despite the success of Utah arrays in producing high quality multi-site recordings in both CNS and PNS, from a long-term stability and FBR perspective these implants are more problematic than other penetrating designs. While as a material silicon is chemically inert and biocompatible, permitting cell growth and not causing particular adverse reactions in tissue over long implantation periods (Bayliss et al., 1999; Shin et al., 2019), its high stiffness leads to poor tissue compatibility when applied to such an invasive format. Utah arrays rupture nerve tissues when implanted for each of the many electrodes it contains, greatly increasing insertion trauma. Moreover, their stiff material body contributes to greater mechanical shear damage around each of the shanks, worsening long-term trauma and FBR. This mechanical damage is severe enough to damage not only the fragile nervous tissue, but also the probes themselves. In the CNS, a tissue less exposed to movement than peripheral nerves, a study determined that 90% of Utah arrays implanted in non-human primates failed over a 6 year chronic implantation period, with most of failures seen within a year of implantation (Barrese et al., 2016). Slanted Utah arrays implanted into nerves cause large amounts of tissue damage, which over long-term implantation periods lead to larger FBR responses and chronic inflammation (Christensen et al., 2014, 2016).

Slanted Utah arrays continue to be of great value as research tools, and show promise for the translation of new therapies to the clinic. Recently, slanted Utah arrays implanted into the median and ulnar nerves of a hand amputee patient. Through stimulation of the nerves, and in combination with muscle recordings, the patient was able to drive and receive sensory feedback from a bionic hand (George et al., 2019). Despite this and other prior translational successes (Clark et al., 2014; Davis et al., 2016; Wendelken et al., 2017), the large degree of trauma and FBR associated with current Utah array designs poses difficult challenges for their widespread clinical use.

While most penetrating electrodes are designed to pierce directly into the endoneurial compartment, certain designs employ a less invasive approach. Slowly penetrating interfascicular nerve electrodes (SPINEs), for example, consist of a nerve cuff incorporating penetrating electrode-equipped flaps over its inner surface, which slowly penetrate into the nerve cross-section after implantation. While piercing into the ECM-rich epineurium, these flaps settle between fascicles and do not pierce the perineurium. By bypassing part of the epineurium, these electrodes achieve a decrease in stimulation threshold and can achieve better selectivity than traditional epineurial cuff designs (Tyler and Durand, 1994, 1997). Although SPINE designs achieve some of the advantages of intrafascicular designs, they can exhibit better long-term stability by localizing fibrosis and inflammation due to FBR to the outer, more robust, layers of the nerve architecture.

On the other end of the spectrum, certain types of nerve neuroprosthetics achieve fascicle recordings by more severely disrupting the tissues of the nerve. Microchannel implants—devices made up of multiple electrode-containing channels designed to each host a subset of the nerve's axons, and more commonly used as regenerative nerve electrodes—have also been used to interface with healthy nerves. Implantation of these devices involves the surgical disruption of the nerve epineurium, followed by the gentle teasing of the axons within into small bundles which are then enclosed within the implant's channels. While the severe implantation trauma caused by the full dissection of the epineurium can lead to axon damage, certain nerves are able to support this implantation procedure (Chew et al., 2013). Such microchannel electrodes have, for example, been implanted in nerves at locations very proximal to the dorsal root ganglia to monitor bladder function in rodents at long-term implantation time points (Chew et al., 2013). However, the implanted nerves experienced a steady loss of axons over a 3 month period, likely a consequence of the implantation trauma combined with subsequent damage caused by the proximity of the implant walls to the fragile axons. Their good recording properties and ease of use have, however, found use for them in *ex vivo* applications such as nerve-on-a-chip platforms (Gribi et al., 2018).

### Regenerative Nerve Electrodes

The last class of implants are regenerative nerve electrodes, which exploit the regenerative capacity of peripheral nerves in order to embed electrodes deeply into nerve tissue. In contrast to the central nervous system, peripheral nerves are capable of undergoing some degree of regeneration when injured. When a nerve is damaged axons past the lesion site degenerate, while axons proximal to the lesion begin regrowing across it in an attempt to reconnect with their distal target. If an implant containing electrodes is introduced into a nerve lesion, these axons will often grow through the implant as part of their regenerative response. This strategy can be used to position large numbers of electrodes throughout the entire cross-section of the nerve and, once the regenerating axons reconnect to their targets at the distal end of the nerve, the array of electrodes can be used stimulate or record the electrical activity of small axon subsets.

While regenerative implants are effective at tightly interfacing large numbers of electrodes with a nerve, they are also associated with the largest amount of implantation trauma. Whether occurring accidentally or purposely induced (in research models), regenerative implants require a nerve lesion to be present. Most commonly this is in the form of a full nerve transection, where the two ends of the nerve are cleanly separated and a gap forms between the two (Navarro et al., 2005; Thompson et al., 2015). Lesioning brings with it large amounts of inflammation and fibrosis which, although initially directed toward guiding axon regeneration and repairing the nerve tissue, can later worsen FBR around the implant.

The first widely used type of regenerative nerve neuroprosthetics to be developed were sieve electrodes. Early sieve electrode designs were fabricated from rigid silicon wafers perforated with an array of holes, each of which contained an electrode (Edell, 1986; Akin et al., 1994; Navarro et al., 1996). Silicon is bioinert as a material, permitting cell growth (Bayliss et al., 1999) and remaining stable for long periods of implantation without causing adverse tissue reactions (Shin et al., 2019). However, the high stiffness of silicon can lead to mechanical damage surrounding silicon implants. Silicon sieves were observed to lead to extensive axon death after implantation and were replaced by more flexible materials such as polyimide in later designs (Shimatani et al., 2003; Ramachandran et al., 2006). Upon implantation into a transected nerve axons would regenerate through the sieve's holes, allowing stimulation or monitoring of their electrical activity. While holes with diameters as small as individual axons (2–10 μm) could theoretically have allowed individual recordings of single regenerated axons, axon regeneration fails through such small holes (Navarro et al., 1998). Instead, holes of 40–65 μm were typically preferred (Navarro et al., 1998). Sieve electrodes implanted into regenerating nerves in rodent models have been successfully used to record gustatory (Shimatani et al., 2003) as well as sensorimotor (Ramachandran et al., 2006) signals weeks after implantation. More recently, additional features such as the incorporation of porous “transit” zones in the center of the sieve and the use of glial derived neurotrophic factor (GDNF) to improve nerve regeneration and survival have been implemented (MacEwan et al., 2016).

Aside from the injury-associated trauma related to the implantation of a regenerative implant, sieve electrodes are also vulnerable to further long-term tissue trauma and FBR. The narrow holes through which axons regenerate become a choke point where trauma and fibrosis can easily develop. Implanted sieve electrodes made of polyimide experience a steady increase in axonal numbers over the first 6 months post-implantation, as all axons slowly regenerate through the implant and into the distal nerve stump. This is, however, followed by a sharp decline in axon numbers at the 12 month time point (Lago et al., 2005). Inflammation, characterized by an accumulation of macrophages around the edges of the sieve holes, appears to peak 6 months into implantation followed by a moderate decline—possibly as the holes become filled with fibroblasts and ECM (Klinge et al., 2001).

A more recent evolution of the sieve electrode design are microchannel electrodes, composed of a solid cylinder of material perforated by an array of long channels, each housing within them one or more electrodes. Microchannel devices exploit the fact that fully transected nerves are capable of regenerating across up to centimeter-long gaps left between the nerve stumps (Deal et al., 2012). Microchannel devices are typically several millimeters in length, and have channels of ~100 μm diameter. While microchannel electrodes might at first look like a longitudinally-extended version of the sieve electrode design, microchannels offer a number of advantages. The walls of the microchannels—made from insulating materials—serve to amplify the weak axon electrical signals, and shield the electrodes from external noise (Fitzgerald et al., 2008). While originally made from polyimide (FitzGerald et al., 2012), more recent microchannel designs are built from stretchable and softer materials such as PDMS (Musick et al., 2015; Lancashire et al., 2016). Microchannel devices have been used to record sensorimotor nerve signals in rodent models (Gore et al., 2015; Srinivasan et al., 2015b), and have been shown to work even in absence of a regeneration target in full amputation models (Srinivasan et al., 2015a).

While many of the same implications for FBR that apply sieve electrodes also apply to microchannel electrodes, microchannels both offer some advantages and face additional challenges. On one hand, microchannel devices are often made from stretchable materials, such as PDMS. These materials allow the channel walls to deform, in contrast to sieve polyimide sieve electrodes which can only bend within the nerve cross-sectional plane. This can minimize the degree of long-term damage caused to the nerve tissue within the channels, decreasing FBR and axon death. Studies have achieved long-term recordings in freely moving animals using microchannel devices for periods 4 months post-implantation (Gore et al., 2015), while only small signals in response to electrical stimulation have been recorded at such chronic timepoint with sieve electrodes (Ramachandran et al., 2006). The channels in microchannel devices are, however, orders of magnitude longer than the holes of a sieve electrode. This creates a need to fully vascularise the tissue within the channels to supply the axons with nutrients and prevent their death, which makes them more vulnerable to the slow build-up of fibrotic tissue during chronic FBR. While axon regeneration can occur in microchannel devices with channels as small as small as 55 μm in diameter (similar to sieve electrode dimensions), optimal axon regeneration is seen when coupled with growth of one or more blood vessel, requiring larger microchannels of 100 μm diameter (Lacour et al., 2009). Once regeneration has occurred, however, the progressive narrowing of the channel diameter as FBR develops over its inner wall leads to the eventual occlusion of these blood vessels, depriving axons of their nutrient supply. By 6 months post-implantation PDMS microchannel devices can develop capsules within the channels 28 μm in thickness which, while still leaving a large portion of the channel diameter unoccluded, lead to the death of the majority of the axons (FitzGerald, 2016).

While sieve and microchannel electrodes are the most distinct regenerative nerve designs, specifically developed to be used in regenerating nerves, many other classes of implantable nerve neuroprosthetics can be used as regenerative devices. Cuff electrodes can be inserted into the gap of a nerve lesion and the nerve allowed to grow through it, rather than wrapping the cuff around a healthy nerve (Lotfi et al., 2011; Stoyanova et al., 2013; Thompson et al., 2015). Regenerative designs ensure that tissue always perfectly surrounds the implant, providing tighter interfacing and higher quality electrical stimulation/recording properties without accidentally excessively compressing the nerve. Similarly, cylindrical conduits implanted into a gap lesion can also be equipped with penetrating implants facing its bore, allowing axons to regrow around them for better interfacing (Musallam et al., 2007; Clements et al., 2013). Thin-film devices can also be incorporated into bridging conduits to split the bore into two or more channels, achieving some of the higher-selectivity benefits provided by microchannel designs (Delgado-Martínez et al., 2017). Although these devices may perform better than their non-regenerative counterparts, the large amount of trauma and subsequent inflammation and FBR are unlikely to be worthwhile trade-off unless a nerve lesion has already occurred.

## Looking Ahead—New Materials and Strategies to Minimize FBR

The development of a foreign body reaction in response to the implantation of materials into nerves has been reported in literature for decades (Kim et al., 1976). Over the years, a wide range of nerve neuroprosthetic designs have been developed and tested. This has provided a wealth of data on the advantages and disadvantages of different designs, including their effect on FBR and tissue trauma. While a lot can be learned from past designs for the development of implants with better long-term stability and tissue compatibility, there has also been an increasing interest in gaining a deeper understanding of the causes and pathways underlying FBR—and ways of directly tackling this reaction (Table 2).

**Table 2:** Summary of factors influencing FBR to implants and strategies employed to minimize it.

**Implant properties and downstream effects influencing FBR**	**Strategies to minimize FBR**
Chemical biocompatibility and material degradation	• Bioinert and long-term stable materials. • Degradable materials that are removed from the body before FBR progresses into its chronic stage.
Biofouling of the implant	• Low fouling materials or coatings at the implant surface.
Implant size and shape	• Microfabrication of smaller implants and use of designs better suited for their nerve environment.
Implant stiffness	• Soft and flexible materials or implant coatings.
Tissue inflammation	• Anti-inflammatory compounds, particularly locally delivering through the implant. • Compounds targeting FBR-specific inflammation.
Fibrosis and capsule formation	• Anti-fibrotic compounds. • Suppressants of vascular growth.

*Note certain factors contributing to FBR have more than one strategy FBR-reduction strategy associated*.

FBR occurs in response to any material that is implanted into the body (Anderson et al., 2008; Salatino et al., 2017). However, certain material properties can exacerbate or decrease FBR. Implantable materials must exhibit good biocompatibility, being chemically inert to tissue to minimize inflammation and cell death (Williams, 2008; Hassler et al., 2011; Mariani et al., 2019), and remain so for the necessary long implantation periods. Even if used materials are chosen to be biocompatible, mechanical factors such as the size and geometry of implanted bodies fabricated from these also has a direct impact on the severity of the FBR. Trauma is intimately tied to FBR, as the inflammation resulting from tissue damage will attract immune cells to the area which can join the reaction against the implant (monocyte/macrophage recruitment step, Figure 1). Different size and shape implants will trigger different amounts of FBR based on the degree of mechanical trauma they cause in when moving in their implanted location. However, even when these variables are eliminated—for example by implanting a mass of spherical implants of different sizes into the peritoneal cavity of rodent models—size appears to directly impact on FBR. Spherical implants of 0.5 mm diameter generate higher degrees of FBR than larger 1.5 mm ones, an effect which is consistent for a wide range of materials (Veiseh et al., 2015). Similarly, shape appears to directly influence FBR, with sharper edges generating more fibrosis than spherical implants (Veiseh et al., 2015). Very small implants—with dimensions smaller than individual cells—such as injectable mesh electrodes seem to generate minimal amounts of FBR, potentially largely bypassing this response by preventing cells (neutrophils, macrophages and fibroblasts in Figure 1) from fully spreading on their surface and becoming activated (Zhou et al., 2017). Interference with cell adhesion can also be achieved through chemical means. Low fouling materials minimize the adsorption of proteins to their surface—the first step leading to FBR—and coating implants with these can greatly diminish FBR (Xie et al., 2018).

Material stiffness also greatly contributes to FBR, with softer materials (closer in stiffness to that of biological tissues) generating less mechanical trauma and therefore less FBR (Lacour et al., 2016; Salatino et al., 2017). However, similarly to implant size, stiffness also appears to play a direct role in guiding FBR independently of the mechanical damaged caused during movement. Nerve implants coated with a range of very low stiffness materials develop less fibrosis and inflammation than those coated with less soft materials (Carnicer-Lombarte et al., 2019). How properties such as size, geometry and stiffness directly guide FBR is not understood. While no specific mechanism has been identified, mechanotransduction (the conversion of mechanical cues into biochemical signals) is thought to play a role. Cells such as macrophages are known to actively interact with their mechanical environment and modulate their inflammatory activity in response (Iskratsch et al., 2014).

Pharmacological therapies also have great potential to target the downstream consequences of FBR. FBR is primarily mediated by inflammation—macrophages respond to the presence of the implant and orchestrate the fibrotic response that leads to its encapsulation. Anti-inflammatory compounds can directly inhibit FBR by dampening this inflammatory response. A decrease in macrophage activity around the implant decreases both their adherence and proliferation around the implant and the recruitment of capsule-forming fibroblasts (Figure 1). Dexamethasone—a commonly used anti-inflammatory corticosteroid drug—has found great success in pacemaker stimulator implants used in the clinic, where it is incorporated into the material surrounding the electrodes and elutes over time into the surrounding tissue to hamper FBR and improve implant life-time and stability (Mond et al., 2014). Dexamethasone has also been tested for control of FBR in nerve neuroprosthetics. Regenerative microchannel implants incorporating dexamethasone show a decrease in the thickness of the fibrotic capsule and avoid the death of axons over time seen in un-treated implants (FitzGerald, 2016). Dexamethasone administered systemically has also been shown to improve the electrical stimulation properties using TIME implants over a 3-month implantation period (Oliva et al., 2019). Despite its effectiveness, dexamethasone hampers all processes mediated by inflammation—including tissue healing. Dexamethasone use can limit tissue healing following implantation trauma and the chronic trauma associated with the indwelling implant, which can be particularly detrimental in regenerative implants relying on the regrowth of nerve tissue into the implant (FitzGerald, 2016). Similar to dexamethasone, other general anti-inflammatory drugs such as aspirin (Malik et al., 2011) and rapamycin (Takahashi et al., 2010) have also been shown effective for the reduction of FBR. However, although the pathway underlying FBR in inflammation has not been characterized, certain mediators are beginning to be identified and provide new pharmacological targets against FBR. CSF1 receptor has been recently identified as playing a unique role in FBR-associated inflammation, with its inhibition using both implant-encapsulated and systemically-delivered small molecule compounds leading to a decrease in macrophage activity and fibrosis around implanted materials without impacting wound healing (Doloff et al., 2017; Farah et al., 2019).

Further downstream in the FBR process, fibrosis can also be targeted as a means to eliminate some of the more severe consequences of FBR (fibrotic encapsulation, the last step of FBR—Figure 1). TGF-β lies at the heart of the fibrotic response in FBR, driving the activation of fibroblasts surrounding the implant into myofibroblasts (Ignotz and Massagué, 1986). Local inhibition of TGF-β through injection of anti-sense oligonucleotides has been shown to decrease capsule formation in subcutaneous implants (Mazaheri et al., 2003). Suppressors of TGF-β are entering the clinic for the management of fibrotic diseases, offering an opportunity for their use in FBR (King et al., 2014). Growth of the fibrotic capsule also requires the extension of new blood vessels into the newly forming tissue. Local scavenging of VEGF around implants, a stimulator of endothelial growth released by anoxic tissue, can inhibit the extension of new blood vessels necessary to support the fibrotic capsule and decrease fibrotic encapsulation (Dondossola et al., 2017).

**Figure 6:**
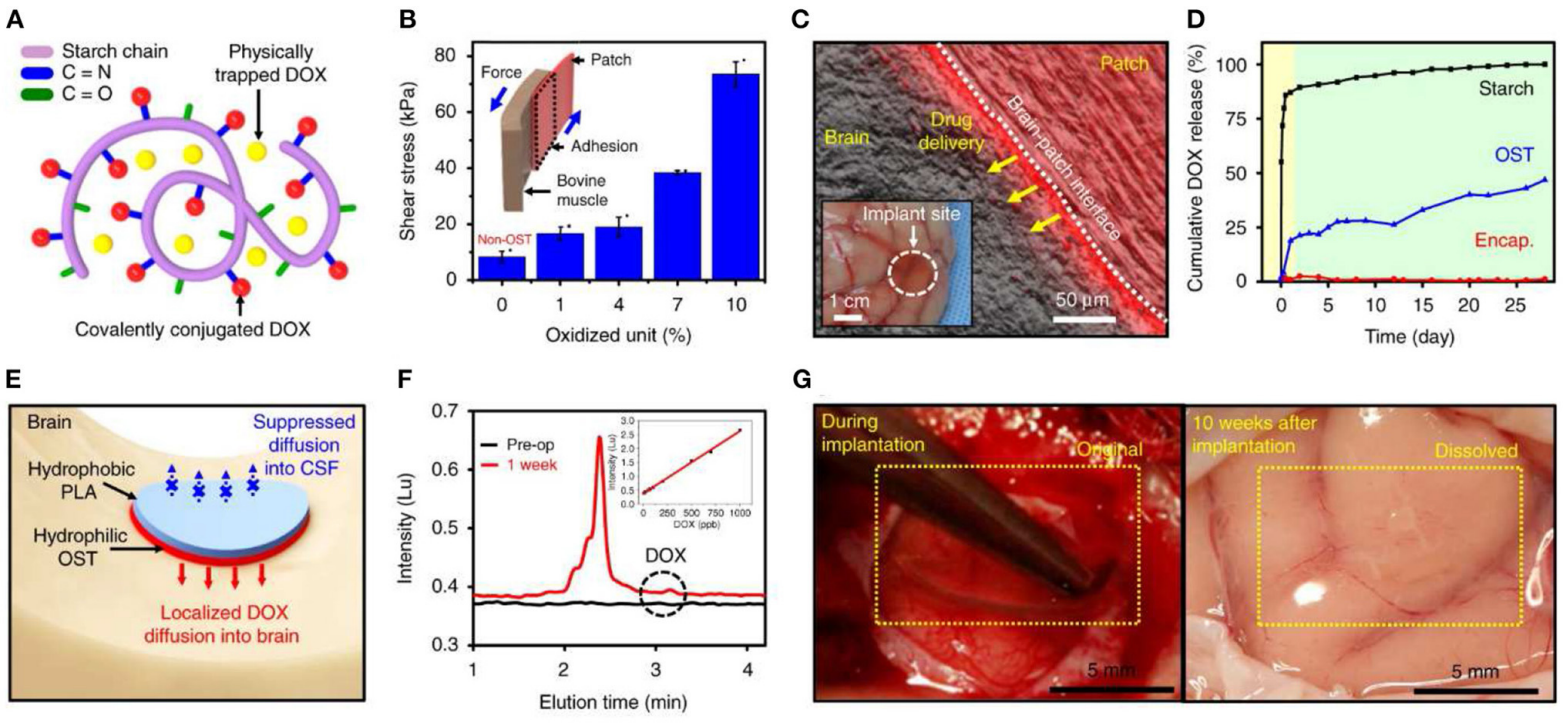
Bioresorbable electronic patch (BEP) implant for controlled drug release. Reproduced under the terms of the Creative Commons Attribution License (Lee et al., 2019). **(A,B)** The patches are fabricated from a drug-loaded (doxorubicin) oxidized starch (OST) reservoir, and an electronics-containing compartment made from Mg (conductor), PLGA (dielectric), and PLA (encapsulation) which is bound to it. **(C)** Wireless control mediates drug delivery into neural tissue. **(D-F)** Doxorubicin release into tissue occurs over a period of days. **(G)** The entire implant becomes fully resorbed within 10 weeks of implantation, leaving no adverse reaction in the nearby tissue.

Apart from surface modification or adjusting the invasiveness of the implants, devices made of biodegradable and bioresorbable materials have been increasingly gathering momentum as a viable class of bioelectronics to minimize the effect of FBR when implanted (Veiseh and Vegas, 2019) (Figure 6). The fibrotic components of FBR develop to chronically-indwelling implants. Biodegradable implants prevent the development of fibrosis by allowing the acute inflammatory phases of FBR to degrade part or the entirety of the implant once its therapeutic role is complete. Biodegradable and bioresorbable devices offer a unique opportunity to simultaneously perform targeted diagnosis and therapy *in vivo* with minimal long-term FBR consequences (Grossen et al., 2017).

For applications where the functions of the implants are only necessary for a predefined period of time, this class of so-called transient electronics employ a layered design (Hwang et al., 2012) where degradable polymeric materials act as an encapsulation layer, with functional inorganic materials such as zinc, magnesium, or silicon enclosed inside. While the active layers can remain long-term *in vivo* (producing a much smaller, lower trauma-inducing device than the original implanted one), they can also themselves be degradable. Not only does the degradable device prevent any need for later surgical extraction once the device lifetime has expired (Mattina et al., 2020), biodegradable devices also provide an alternative route for the immune system to process the device and lead to a much lower degree of FBR. Both the degradation time and rate of degradation can be adjusted by modulating the thickness between the active and encapsulation layers, allowing the devices to remain *in vivo* from days, weeks, to months depending on the applications (Choi et al., 2016).

Rogers and colleagues have designed and fabricated a class of biodegradable sensors to monitor relevant physiological parameters such as cerebral temperature and oxygenation (Bai et al., 2019), spatiotemporal neural activities in the cerebral cortex (Yu et al., 2016), and intracranial pressure (Kang et al., 2016). Biodegradable therapeutic devices such as electronic stents for controlled drug releases for endovascular diseases (Son et al., 2015), or wireless tissue heating for infection treatment (Tao et al., 2014) both demonstrated the high specificity and efficacy of targeted implantable devices while avoiding the negative tissue encapsulation due to FBR.

However, some studies still reported moderate degrees of FBR at the implantation sites of biodegradable bioelectronics (Xue et al., 2014). These studies postulated that the FBR might be due to incomplete degradation of the polymeric materials, and/or the accumulation of debris after the materials are degraded. As a result, careful design and modulating between the active/encapsulating layers of the biodegradable electronics is essential to the success of mitigating FBR in this class of implantable electronics.

## The Future of Nerve Neuroprosthetics

Despite its unavoidable presence, FBR has until recently been an acceptable side effect of the implantation of nerve neuroprosthetics. In the clinic, nerve cuffs have bypassed its effect by delivering larger stimulating currents to entire nerves, while in the research environment shorter implantation time points have allowed the development of implantable technologies without allowing fibrosis to develop. However, nerve neuroprosthetics continue to evolve with advances in knowledge of materials and manufacturing techniques, leading to higher resolution designs requiring more intimate interfacing with neuronal tissues. Together with a growing interest in the translation of these technologies into human clinical use (Wendelken et al., 2017; D'Anna et al., 2019; Petrini et al., 2019), FBR has become a dominant problem that needs to be actively addressed at the implant design stage.

Since its infancy, the field of nerve neuroprosthetics has overall transitioned from stiff materials, well suited for traditional microfabrication techniques, to flexible devices. While cuffs have been the predominant choice for widespread clinical stimulation applications, newer ultraflexible devices such as the TIME are seeing great success in humans (D'Anna et al., 2019; Petrini et al., 2019). The success of these devices can serve to inspire the design of new flexible nerve implant designs and drive a push for the translation of existing ones toward human use. CNS neural interfaces are experiencing a similar shift toward soft/flexible designs (Salatino et al., 2017). Low FBR designs developed for brain applications may be adapted for use in peripheral nerves, such as injectable mesh electrodes (Zhou et al., 2017) or ultraflexible non-penetrating transistor arrays (Khodagholy et al., 2013). Neural tissue is some of the softest in the body (Cox and Erler, 2011), and remains orders of magnitude softer than many materials commonly regarded as soft (Lacour et al., 2016), leaving ample room for the improvement of current soft designs into even more biocompatible and FBR-minimizing ones.

A transition to soft nerve neuroprosthetics will bring with it fabrication challenges. The field of neuroprosthetics has greatly benefitted from microfabrication technology used in the semiconductor industry, which does not easily translate to soft or flexible substrates. This becomes particularly relevant as implant designs become smaller in scale and increasingly elaborate in order to record signals from small subsets of axons within a nerve. A range of strategies are being developed with this objective, such as the use of stiff but also thin and therefore flexible materials (Khodagholy et al., 2015), the engineering of defects in stiff materials to render them deformable (Vachicouras et al., 2017), or the encapsulation of electronics in soft materials (Liu et al., 2019).

An alternative—or more likely a complementary—strategy for future nerve neuroprosthetic designs will be the incorporation of compounds or materials that actively target FBR. Several FBR-targeting compounds are already progressing toward use in humans (King et al., 2014; Farah et al., 2019), and as our understanding of FBR continues to evolve this repertoire will likely continue to expand. Future nerve neuroprosthetics will likely be multimodal, containing not only electrode arrays to electrically communicate with tissue, but also mechanisms to chemically interact with it in the form of FBR-modulating compounds. The delivery of these compounds may occur through engineered structures such as microfluidic channels, or provided through material-oriented solutions such as immobilization of compounds on their surface or encapsulation within materials. The field of surgical nerve repair already employs implants actively modulating biological processes (Carvalho et al., 2019), and may be a useful source for the development of new-generation nerve neuroprosthetics. Other novel strategies, such as the incorporation of cells or tissue within devices to transform them into living implants, interacting with and integrating into the host (Rochford et al., 2020), may also provide unique avenues to address FBR. While the inclusion of these additional modalities introduces further challenges for the manufacturing of neuroprosthetics, the therapeutic potential of implantable devices capable of stable, fine resolution recording and stimulation over decades far outweigh the costs.

## Data Availability Statement

The original contributions generated for the study are included in the article/supplementary material, further inquiries can be directed to the corresponding author/s.

## Author Contributions

AC-L and DB designed the paper and analyzed the literature. AC-L and S-TC wrote the paper. GM and DB revised the paper. All the authors read and approved the manuscript.

## Conflict of Interest

The authors declare that the research was conducted in the absence of any commercial or financial relationships that could be construed as a potential conflict of interest.

## Abbreviations

CNS: Central nervous system
DAPI: 4′,6-diamidino-2-phenylindole
ECM: Extracellular matrix
FBGC: Foreign body giant cell
FBR: Foreign body reaction
FINE: Flat interface nerve electrode
IL-1β: Interleukin 1 beta
LIFE: Longitudinally implanted intrafascicular electrode
NF: Neurofilament
PDGF: Platelet-derived growth factor
PDMS –: Polydimethylsiloxane
PET: Polyethylene terephthalate
PLA: Poly(lactic acid)
PLGA: Poly(lactic-co-glycolic acid)
PMMA: Poly(methyl methacrylate)
PNS: Peripheral nervous system
ROS: Reactive oxygen species
SEM: Scanning electron microscope
SPINE: Slowly penetrating interfascicular nerve electrode
TGF-β: Transforming growth factor beta
TIME: Transverse intrafascicular multichannel electrode
TNFα: Tumor necrosis factor alpha
VEGF: Vascular endothelial growth factor.
